# EDUCATIONAL STRATEGIES TO REDUCE CHILDREN’S EXCESSIVE EXPOSURE TO RADIOLOGICAL EXAMS

**DOI:** 10.1590/1984-0462/;2017;35;2;00018

**Published:** 2017

**Authors:** Henrique Manoel Lederman

**Affiliations:** a Departamento de Diagnóstico por Imagem da Escola Paulista de Medicina, Universidade Federal de São Paulo (UNIFESP), São Paulo, SP, Brasil.

Bernardo & Almeida, from Pontifícia Universidade Católica de São Paulo (PUC-SP), *campus* Sorocaba, are publishing an interesting study in this issue, regarding a problem that is little discussed in our field: strategies to reduce the exposure of children to excessive radiation during radiological examinations.^1^

The program adopted by the authors to reduce the children’s exposure to radiation is based on an international program used all over the world, developed to improve technical standards in pediatric examinations, especially in general hospitals. In these facilities, most technicians or technologists are not specifically trained to work with children, and usually do not use the most adequate parameters for pediatric patients in more sophisticated examinations, like computerized tomography.

The idea of using radioprotection came from the United States a few years ago. If, on one hand, the program can raise awareness among health professionals and families regarding the children’s exposure to radiation, on the other it may promote a discussion about the triggering of family anxiety, and a possible judicialization of health radiology professionals. As to the latter, it is worth mentioning how difficult it is for a lay person to interpret the doses of radiation and the corresponding dosage registered in the equipment with the real amount to which the children have been exposed. Because of the concerns presented herein, the use of radioprotection was temporarily suspended in the United States of America. To the suspension of the program, the action of not causing more difficulties to initiate or suspend examinations, was taken into consideration.

In our field, I believe that, the training of health professionals for the introduction of this initiative is not available yet, and the public is not properly educated to interpret numbers/doses.

The consensus today is that every radiological examination should be performed with the lowest possible dose of radiation, using the best technique and maximum quality. The indication of the exam should be made carefully, always considering the risk and the benefit, as well as the possibility that an examination result might change the course of medical conduct. On the basis of this principle, we are always working with radioprotection.

## References

[1] Bernardo MO, de Almeida FA (2017). Campanha e carteira de radioproteção: estratégias educativas que reduzem a exposição excessiva de crianças a exames radiológicos. Rev Paul Pediatr.

